# Azido­(benzoyl­acetonato-κ^2^ *O*,*O*′)[1-phenyl-3-(2-pyridylmethyl­imino)but-1-en-1-olato-κ^3^ *N*,*N*′,*O*]cobalt(III)

**DOI:** 10.1107/S1600536810003338

**Published:** 2010-02-03

**Authors:** Amitabha Datta, Ming-Han Sie, Jui-Hsien Huang, Hon Man Lee

**Affiliations:** a National Changhua University of Education, Department of Chemistry, Changhua, Taiwan 50058

## Abstract

In the title complex, [Co(C_16_H_15_N_2_O)(C_10_H_9_O_2_)(N_3_)], the Co^II^ atom adopts an octa­hedral coordination geometry by a tridentate Schiff base, a bidentate benzoyl­acetonate and an azide ligand. The imine N atom of the tridentate ligand is *trans* to the benzoyl O atom of the bidentate ligand and the azide ligand is *trans* to the acetyl O atom of the bidentate ligand. Non-classical intra­molecular C_ar­yl_—H⋯O hydrogen bonds are present in the structure.

## Related literature

For the preparation of the ligand, see: Ray *et al.* (2009 ▶). For the crystal structure of a related complex, see: Clearfield *et al.* (1978 ▶).
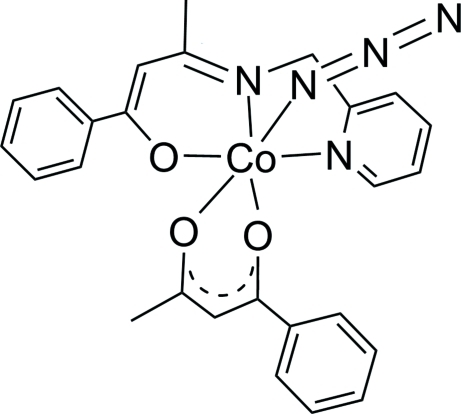


## Experimental

### 

#### Crystal data

[Co(C_16_H_15_N_2_O)(C_10_H_9_O_2_)(N_3_)]
*M* *_r_* = 513.43
Monoclinic, 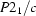

*a* = 14.029 (3) Å
*b* = 14.386 (3) Å
*c* = 12.423 (3) Å
β = 102.752 (6)°
*V* = 2445.4 (9) Å^3^
*Z* = 4
Mo *K*α radiation
μ = 0.74 mm^−1^
*T* = 298 K
0.50 × 0.40 × 0.30 mm

#### Data collection

Bruker SMART APEXII diffractometer
Absorption correction: multi-scan (*SADABS*; Sheldrick, 1996 ▶) *T*_min_ = 0.709, *T*_max_ = 0.809
29548 measured reflections
5064 independent reflections
2004 reflections with *I* > 2σ(*I*)
*R*_int_ = 0.143

#### Refinement

*R*[*F* ^2^ > 2σ(*F* ^2^)] = 0.056
*wR*(*F* ^2^) = 0.147
*S* = 0.88
5064 reflections
319 parameters
1 restraint
H-atom parameters constrained
Δρ_max_ = 0.52 e Å^−3^
Δρ_min_ = −0.29 e Å^−3^

### 

Data collection: *APEX2* (Bruker, 2007 ▶); cell refinement: *SAINT* (Bruker, 2007 ▶); data reduction: *SAINT*; program(s) used to solve structure: *SHELXS97* (Sheldrick, 2008 ▶); program(s) used to refine structure: *SHELXL97* (Sheldrick, 2008 ▶); molecular graphics: *SHELXTL* (Sheldrick, 2008 ▶); software used to prepare material for publication: *DIAMOND* (Brandenburg, 1999 ▶).

## Supplementary Material

Crystal structure: contains datablocks I, global. DOI: 10.1107/S1600536810003338/pv2252sup1.cif

Structure factors: contains datablocks I. DOI: 10.1107/S1600536810003338/pv2252Isup2.hkl

Additional supplementary materials:  crystallographic information; 3D view; checkCIF report

## Figures and Tables

**Table 1:** Hydrogen-bond geometry (Å, °)

*D*—H⋯*A*	*D*—H	H⋯*A*	*D*⋯*A*	*D*—H⋯*A*
C1—H1⋯O1	0.93	2.38	2.699 (6)	100
C16—H16⋯O2	0.93	2.41	2.917 (6)	114
C26—H26⋯O2	0.93	2.34	2.661 (5)	100

**Fig. 1.**
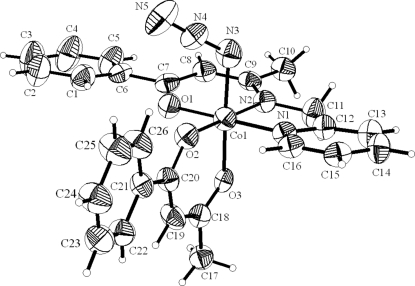
The structure of the title complex, showing 35% displacement ellipsoids for non-H atoms. The H atoms are dipicted by circles of an arbitrary radius.

## supplementary crystallographic information

### Comment

The title complex, (I), was prepared by a one-pot reaction between an unpurified batch of the ligand precursor, (1*Z*,3*E*)-3-((pyridin-2-yl)methylimino)-1-phenylbut-1-en-1-ol, sodium azide, and cobalt acetate tetrahydrate in methanol. The complex consists of a tridentate Schiff base ligand, a benzoylacetonate ligand, and an azide ligand. The Co atom adopts an octahedral geometry. The presence of the bidentate ligand is due to the remaining benzolylacetone in the unpurified batch of the tridenatate ligand precursor. Non-classical intramolecular hydrogen bonds of the type C_aryl_—H···O are present in the structure.

The crystal structure of cobalt azido complex with Schiff base ligand has been reported (Clearfield *et al.*, 1978).

### Experimental

The tridentate Schiff ligand precursor, (1*Z*,3*E*)-3-((pyridin-2-yl)methylimino)-1-phenylbut-1-en-1-ol, was prepared according to the literature procedure (Ray *et al.*, 2009). To an unpurified batch of the ligand precursor (*ca* 2.0 mmol), a methanolic solution (20 ml) of cobalt acetate tetrahydrate (0.249 g, 1.0 mmol) was added, followed by the addition, with constant stirring, of a solution of sodium azide (0.065 g, 1.0 mmol) in minimum volume of water/methanol mixture. The final solution was kept at room temperature yielding brown square-shaped crystals suitable for X-ray diffraction after a few days. Crystals were isolated by filtration and were air-dried.

### Refinement

All the H atoms were positioned geometrically and refined as riding atoms, with C—H = 0.93, 0.96 and 0.97, Å for aryl, methyl and methylene type H-atoms, respectively, while *U_iso_*(H) = 1.5 *U_eq_* (C) for the methyl and 1.2 *U_eq_* (C) for all the other H atoms.

### Crystal data

**Table:**

[Co(C_16_H_15_N_2_O)(C_10_H_9_O_2_)(N_3_)]	*F*(000) = 1064
*M**_r_* = 513.43	*D*_x_ = 1.395 Mg m^−^^3^
Monoclinic, *P*2_1_/*c*	Mo *K*α radiation, λ = 0.71073 Å
Hall symbol: -P 2ybc	Cell parameters from 715 reflections
*a* = 14.029 (3) Å	θ = 2.2–19.8°
*b* = 14.386 (3) Å	µ = 0.74 mm^−^^1^
*c* = 12.423 (3) Å	*T* = 298 K
β = 102.752 (6)°	Square prism, brown
*V* = 2445.4 (9) Å^3^	0.50 × 0.40 × 0.30 mm
*Z* = 4	

### Data collection

**Table:**

Bruker SMART APEXII diffractometer	5064 independent reflections
Radiation source: fine-focus sealed tube	2004 reflections with *I* > 2σ(*I*)
graphite	*R*_int_ = 0.143
ω scans	θ_max_ = 26.5°, θ_min_ = 2.1°
Absorption correction: multi-scan (*SADABS*; Sheldrick, 1996)	*h* = −17→17
*T*_min_ = 0.709, *T*_max_ = 0.809	*k* = −18→17
29548 measured reflections	*l* = −15→15

### Refinement

**Table:**

Refinement on *F*^2^	Secondary atom site location: difference Fourier map
Least-squares matrix: full	Hydrogen site location: inferred from neighbouring sites
*R*[*F*^2^ > 2σ(*F*^2^)] = 0.056	H-atom parameters constrained
*wR*(*F*^2^) = 0.147	*w* = 1/[σ^2^(*F*_o_^2^) + (0.0651*P*)^2^] where *P* = (*F*_o_^2^ + 2*F*_c_^2^)/3
*S* = 0.88	(Δ/σ)_max_ = 0.001
5064 reflections	Δρ_max_ = 0.52 e Å^−^^3^
319 parameters	Δρ_min_ = −0.29 e Å^−^^3^
1 restraint	Extinction correction: *SHELXL97* (Sheldrick, 2008), Fc^*^=kFc[1+0.001xFc^2^λ^3^/sin(2θ)]^-1/4^
Primary atom site location: structure-invariant direct methods	Extinction coefficient: 0.0029 (4)

### Special details

**Table:**

Geometry. All e.s.d.'s (except the e.s.d. in the dihedral angle between two l.s. planes) are estimated using the full covariance matrix. The cell e.s.d.'s are taken into account individually in the estimation of e.s.d.'s in distances, angles and torsion angles; correlations between e.s.d.'s in cell parameters are only used when they are defined by crystal symmetry. An approximate (isotropic) treatment of cell e.s.d.'s is used for estimating e.s.d.'s involving l.s. planes.
Refinement. Refinement of *F*^2^ against ALL reflections. The weighted *R*-factor *wR* and goodness of fit *S* are based on *F*^2^, conventional *R*-factors *R* are based on *F*, with *F* set to zero for negative *F*^2^. The threshold expression of *F*^2^ > σ(*F*^2^) is used only for calculating *R*-factors(gt) *etc*. and is not relevant to the choice of reflections for refinement. *R*-factors based on *F*^2^ are statistically about twice as large as those based on *F*, and *R*- factors based on ALL data will be even larger.

### Fractional atomic coordinates and isotropic or equivalent isotropic displacement parameters (Å^2^)

**Table:**

	*x*	*y*	*z*	*U*_iso_*/*U*_eq_	
C1	0.7324 (4)	1.1726 (4)	0.4878 (4)	0.0851 (15)	
H1	0.7583	1.1374	0.4383	0.102*	
C2	0.7474 (4)	1.2670 (5)	0.4919 (5)	0.112 (2)	
H2	0.7820	1.2952	0.4451	0.135*	
C3	0.7099 (5)	1.3205 (5)	0.5676 (6)	0.130 (2)	
H3	0.7217	1.3841	0.5736	0.156*	
C4	0.6557 (5)	1.2776 (5)	0.6323 (5)	0.125 (2)	
H4	0.6281	1.3128	0.6804	0.150*	
C5	0.6418 (4)	1.1841 (4)	0.6269 (4)	0.0926 (16)	
H5	0.6058	1.1564	0.6727	0.111*	
C6	0.6800 (3)	1.1285 (4)	0.5547 (4)	0.0659 (12)	
C7	0.6688 (3)	1.0260 (3)	0.5500 (3)	0.0608 (12)	
C8	0.6028 (3)	0.9800 (3)	0.5984 (3)	0.0633 (12)	
H8	0.5625	1.0167	0.6311	0.076*	
C9	0.5903 (3)	0.8841 (3)	0.6033 (3)	0.0575 (12)	
C10	0.5208 (3)	0.8478 (3)	0.6717 (4)	0.0714 (13)	
H10B	0.4919	0.8993	0.7021	0.107*	
H10A	0.5562	0.8097	0.7306	0.107*	
H10C	0.4703	0.8116	0.6257	0.107*	
C11	0.6247 (4)	0.7255 (3)	0.5649 (4)	0.0812 (14)	
H11A	0.5555	0.7113	0.5519	0.097*	
H11B	0.6553	0.7054	0.6390	0.097*	
C12	0.6696 (3)	0.6748 (3)	0.4822 (4)	0.0627 (12)	
C13	0.6583 (3)	0.5820 (4)	0.4625 (4)	0.0775 (14)	
H13	0.6218	0.5464	0.5012	0.093*	
C14	0.7015 (3)	0.5409 (3)	0.3845 (4)	0.0752 (14)	
H14	0.6948	0.4775	0.3701	0.090*	
C15	0.7548 (3)	0.5966 (4)	0.3286 (4)	0.0683 (13)	
H15	0.7843	0.5711	0.2753	0.082*	
C16	0.7636 (3)	0.6887 (4)	0.3523 (3)	0.0634 (12)	
H16	0.7996	0.7254	0.3141	0.076*	
C17	0.9868 (3)	0.8414 (3)	0.7072 (3)	0.0724 (13)	
H17A	0.9691	0.8851	0.7578	0.109*	
H17B	1.0531	0.8523	0.7017	0.109*	
H17C	0.9810	0.7793	0.7334	0.109*	
C18	0.9201 (3)	0.8529 (3)	0.5958 (4)	0.0616 (12)	
C19	0.9564 (3)	0.8776 (3)	0.5056 (4)	0.0744 (14)	
H19	1.0235	0.8869	0.5180	0.089*	
C20	0.9042 (3)	0.8899 (3)	0.3995 (4)	0.0575 (11)	
C21	0.9506 (3)	0.9039 (3)	0.3039 (4)	0.0612 (12)	
C22	1.0523 (3)	0.9007 (3)	0.3166 (4)	0.0705 (13)	
H22	1.0925	0.8957	0.3867	0.085*	
C23	1.0931 (4)	0.9051 (3)	0.2247 (5)	0.0862 (16)	
H23	1.1606	0.9015	0.2338	0.103*	
C24	1.0358 (4)	0.9147 (4)	0.1210 (5)	0.0917 (17)	
H24	1.0638	0.9179	0.0599	0.110*	
C25	0.9365 (4)	0.9193 (4)	0.1087 (4)	0.0953 (17)	
H25	0.8967	0.9252	0.0385	0.114*	
C26	0.8941 (4)	0.9153 (3)	0.2001 (4)	0.0772 (14)	
H26	0.8266	0.9206	0.1904	0.093*	
Co1	0.72505 (4)	0.85915 (4)	0.46535 (5)	0.0621 (3)	
N1	0.7223 (2)	0.7297 (2)	0.4290 (3)	0.0593 (9)	
N2	0.6382 (2)	0.8246 (3)	0.5547 (3)	0.0585 (9)	
N3	0.6147 (3)	0.8765 (3)	0.3428 (3)	0.0777 (12)	
N4	0.6194 (3)	0.9354 (3)	0.2769 (4)	0.0749 (11)	
N5	0.6216 (4)	0.9907 (3)	0.2103 (4)	0.1157 (17)	
O1	0.7286 (2)	0.9854 (2)	0.4994 (2)	0.0706 (9)	
O2	0.8099 (2)	0.88660 (19)	0.3693 (2)	0.0676 (8)	
O3	0.8291 (2)	0.83817 (19)	0.5923 (2)	0.0642 (8)	

### Atomic displacement parameters (Å^2^)

**Table:**

	*U*^11^	*U*^22^	*U*^33^	*U*^12^	*U*^13^	*U*^23^
C1	0.084 (4)	0.069 (4)	0.106 (4)	0.009 (3)	0.031 (3)	0.008 (3)
C2	0.132 (5)	0.076 (5)	0.140 (6)	0.001 (4)	0.054 (4)	0.025 (4)
C3	0.165 (7)	0.072 (5)	0.164 (7)	−0.001 (4)	0.061 (6)	−0.006 (5)
C4	0.185 (7)	0.068 (5)	0.140 (6)	0.019 (4)	0.076 (5)	0.001 (4)
C5	0.127 (5)	0.069 (4)	0.090 (4)	0.013 (3)	0.043 (3)	0.005 (3)
C6	0.059 (3)	0.069 (4)	0.067 (3)	0.016 (3)	0.009 (2)	0.007 (3)
C7	0.055 (3)	0.069 (4)	0.059 (3)	0.016 (2)	0.014 (2)	0.005 (2)
C8	0.056 (3)	0.076 (4)	0.063 (3)	0.009 (2)	0.023 (2)	0.000 (2)
C9	0.045 (2)	0.076 (4)	0.053 (3)	0.002 (2)	0.013 (2)	0.005 (2)
C10	0.063 (3)	0.091 (4)	0.067 (3)	−0.001 (2)	0.029 (2)	0.001 (3)
C11	0.094 (4)	0.068 (4)	0.096 (4)	−0.002 (3)	0.051 (3)	0.003 (3)
C12	0.068 (3)	0.063 (3)	0.062 (3)	−0.002 (2)	0.025 (2)	−0.005 (3)
C13	0.095 (4)	0.075 (4)	0.068 (3)	−0.007 (3)	0.031 (3)	−0.003 (3)
C14	0.098 (4)	0.061 (3)	0.069 (3)	−0.008 (3)	0.023 (3)	−0.005 (3)
C15	0.072 (3)	0.074 (4)	0.060 (3)	0.009 (3)	0.015 (3)	−0.008 (3)
C16	0.061 (3)	0.071 (4)	0.061 (3)	0.007 (2)	0.018 (2)	0.002 (3)
C17	0.075 (3)	0.074 (4)	0.065 (3)	−0.002 (2)	0.009 (3)	0.003 (2)
C18	0.069 (3)	0.054 (3)	0.065 (3)	0.005 (2)	0.021 (3)	−0.003 (2)
C19	0.065 (3)	0.092 (4)	0.066 (3)	−0.014 (3)	0.015 (3)	0.003 (3)
C20	0.057 (3)	0.056 (3)	0.065 (3)	−0.004 (2)	0.025 (2)	−0.006 (2)
C21	0.062 (3)	0.067 (3)	0.060 (3)	−0.007 (2)	0.025 (3)	−0.001 (2)
C22	0.068 (3)	0.088 (4)	0.059 (3)	−0.002 (2)	0.021 (3)	0.011 (3)
C23	0.072 (4)	0.096 (4)	0.102 (5)	0.003 (3)	0.041 (4)	0.009 (3)
C24	0.078 (4)	0.131 (5)	0.072 (4)	−0.010 (3)	0.028 (3)	0.009 (3)
C25	0.082 (4)	0.140 (5)	0.067 (4)	−0.019 (3)	0.022 (3)	0.003 (3)
C26	0.069 (3)	0.116 (4)	0.050 (3)	−0.012 (3)	0.019 (3)	0.006 (3)
Co1	0.0620 (4)	0.0668 (5)	0.0648 (4)	0.0038 (3)	0.0301 (3)	0.0043 (3)
N1	0.061 (2)	0.065 (3)	0.055 (2)	0.0059 (18)	0.0195 (19)	0.0001 (18)
N2	0.056 (2)	0.072 (3)	0.052 (2)	0.0026 (18)	0.0216 (18)	0.0094 (18)
N3	0.080 (3)	0.089 (3)	0.074 (3)	−0.002 (2)	0.038 (2)	0.005 (2)
N4	0.090 (3)	0.074 (3)	0.067 (3)	0.023 (2)	0.031 (2)	0.0026 (19)
N5	0.177 (5)	0.088 (4)	0.088 (4)	0.037 (3)	0.043 (3)	0.022 (3)
O1	0.0675 (19)	0.064 (2)	0.090 (2)	0.0054 (15)	0.0381 (18)	0.0053 (16)
O2	0.066 (2)	0.076 (2)	0.070 (2)	0.0016 (16)	0.0349 (16)	0.0089 (16)
O3	0.060 (2)	0.078 (2)	0.0572 (19)	0.0035 (16)	0.0173 (15)	0.0026 (15)

### Geometric parameters (Å, °)

**Table:**

C1—C2	1.372 (7)	C15—H15	0.9300
C1—C6	1.380 (6)	C16—N1	1.355 (5)
C1—H1	0.9300	C16—H16	0.9300
C2—C3	1.404 (7)	C17—C18	1.498 (6)
C2—H2	0.9300	C17—H17A	0.9600
C3—C4	1.370 (8)	C17—H17B	0.9600
C3—H3	0.9300	C17—H17C	0.9600
C4—C5	1.359 (7)	C18—O3	1.285 (5)
C4—H4	0.9300	C18—C19	1.376 (6)
C5—C6	1.394 (6)	C19—C20	1.370 (6)
C5—H5	0.9300	C19—H19	0.9300
C6—C7	1.484 (6)	C20—O2	1.295 (5)
C7—O1	1.294 (4)	C20—C21	1.488 (5)
C7—C8	1.378 (5)	C21—C26	1.366 (6)
C8—C9	1.393 (6)	C21—C22	1.402 (6)
C8—H8	0.9300	C22—C23	1.387 (6)
C9—N2	1.314 (5)	C22—H22	0.9300
C9—C10	1.521 (5)	C23—C24	1.367 (6)
C10—H10B	0.9600	C23—H23	0.9300
C10—H10A	0.9600	C24—C25	1.370 (6)
C10—H10C	0.9600	C24—H24	0.9300
C11—N2	1.447 (5)	C25—C26	1.395 (6)
C11—C12	1.507 (6)	C25—H25	0.9300
C11—H11A	0.9700	C26—H26	0.9300
C11—H11B	0.9700	Co1—O1	1.862 (3)
C12—N1	1.350 (5)	Co1—N2	1.887 (3)
C12—C13	1.360 (6)	Co1—O2	1.903 (3)
C13—C14	1.383 (6)	Co1—N1	1.915 (4)
C13—H13	0.9300	Co1—O3	1.922 (3)
C14—C15	1.382 (6)	Co1—N3	1.933 (4)
C14—H14	0.9300	N3—N4	1.190 (5)
C15—C16	1.358 (6)	N4—N5	1.153 (5)
			
C2—C1—C6	122.0 (5)	C18—C17—H17C	109.5
C2—C1—H1	119.0	H17A—C17—H17C	109.5
C6—C1—H1	119.0	H17B—C17—H17C	109.5
C1—C2—C3	119.4 (6)	O3—C18—C19	123.9 (4)
C1—C2—H2	120.3	O3—C18—C17	115.2 (4)
C3—C2—H2	120.3	C19—C18—C17	120.9 (4)
C4—C3—C2	119.0 (6)	C20—C19—C18	127.0 (4)
C4—C3—H3	120.5	C20—C19—H19	116.5
C2—C3—H3	120.5	C18—C19—H19	116.5
C5—C4—C3	120.6 (6)	O2—C20—C19	124.7 (4)
C5—C4—H4	119.7	O2—C20—C21	111.9 (4)
C3—C4—H4	119.7	C19—C20—C21	123.4 (4)
C4—C5—C6	121.9 (5)	C26—C21—C22	118.3 (4)
C4—C5—H5	119.1	C26—C21—C20	120.3 (4)
C6—C5—H5	119.1	C22—C21—C20	121.3 (4)
C1—C6—C5	117.1 (5)	C23—C22—C21	120.0 (4)
C1—C6—C7	120.0 (4)	C23—C22—H22	120.0
C5—C6—C7	122.9 (5)	C21—C22—H22	120.0
O1—C7—C8	124.4 (4)	C24—C23—C22	121.2 (5)
O1—C7—C6	113.1 (4)	C24—C23—H23	119.4
C8—C7—C6	122.4 (4)	C22—C23—H23	119.4
C7—C8—C9	126.7 (4)	C23—C24—C25	118.8 (5)
C7—C8—H8	116.7	C23—C24—H24	120.6
C9—C8—H8	116.7	C25—C24—H24	120.6
N2—C9—C8	122.9 (4)	C24—C25—C26	120.9 (5)
N2—C9—C10	119.2 (4)	C24—C25—H25	119.6
C8—C9—C10	117.9 (4)	C26—C25—H25	119.6
C9—C10—H10B	109.5	C21—C26—C25	120.7 (5)
C9—C10—H10A	109.5	C21—C26—H26	119.6
H10B—C10—H10A	109.5	C25—C26—H26	119.6
C9—C10—H10C	109.5	O1—Co1—N2	96.43 (14)
H10B—C10—H10C	109.5	O1—Co1—O2	87.17 (12)
H10A—C10—H10C	109.5	N2—Co1—O2	176.08 (15)
N2—C11—C12	109.6 (4)	O1—Co1—N1	179.28 (14)
N2—C11—H11A	109.8	N2—Co1—N1	84.29 (16)
C12—C11—H11A	109.8	O2—Co1—N1	92.11 (13)
N2—C11—H11B	109.8	O1—Co1—O3	89.39 (12)
C12—C11—H11B	109.8	N2—Co1—O3	87.02 (13)
H11A—C11—H11B	108.2	O2—Co1—O3	94.59 (12)
N1—C12—C13	122.7 (4)	N1—Co1—O3	90.73 (13)
N1—C12—C11	114.0 (4)	O1—Co1—N3	91.70 (16)
C13—C12—C11	123.2 (4)	N2—Co1—N3	89.32 (15)
C12—C13—C14	119.6 (4)	O2—Co1—N3	89.01 (14)
C12—C13—H13	120.2	N1—Co1—N3	88.23 (16)
C14—C13—H13	120.2	O3—Co1—N3	176.28 (14)
C13—C14—C15	118.3 (5)	C12—N1—C16	117.1 (4)
C13—C14—H14	120.9	C12—N1—Co1	116.0 (3)
C15—C14—H14	120.9	C16—N1—Co1	126.8 (3)
C16—C15—C14	119.4 (4)	C9—N2—C11	120.8 (3)
C16—C15—H15	120.3	C9—N2—Co1	124.1 (3)
C14—C15—H15	120.3	C11—N2—Co1	115.1 (3)
N1—C16—C15	122.9 (4)	N4—N3—Co1	118.5 (3)
N1—C16—H16	118.5	N5—N4—N3	177.6 (6)
C15—C16—H16	118.5	C7—O1—Co1	124.2 (3)
C18—C17—H17A	109.5	C20—O2—Co1	124.5 (3)
C18—C17—H17B	109.5	C18—O3—Co1	124.8 (3)
H17A—C17—H17B	109.5		
			
C6—C1—C2—C3	1.0 (9)	N2—Co1—N1—C12	−3.3 (3)
C1—C2—C3—C4	−2.7 (10)	O2—Co1—N1—C12	178.3 (3)
C2—C3—C4—C5	2.8 (11)	O3—Co1—N1—C12	83.6 (3)
C3—C4—C5—C6	−1.2 (10)	N3—Co1—N1—C12	−92.8 (3)
C2—C1—C6—C5	0.5 (7)	N2—Co1—N1—C16	173.3 (3)
C2—C1—C6—C7	−177.8 (5)	O2—Co1—N1—C16	−5.2 (3)
C4—C5—C6—C1	−0.4 (8)	O3—Co1—N1—C16	−99.8 (3)
C4—C5—C6—C7	177.9 (5)	N3—Co1—N1—C16	83.8 (3)
C1—C6—C7—O1	14.6 (6)	C8—C9—N2—C11	178.2 (4)
C5—C6—C7—O1	−163.7 (4)	C10—C9—N2—C11	0.6 (6)
C1—C6—C7—C8	−167.4 (4)	C8—C9—N2—Co1	−3.5 (6)
C5—C6—C7—C8	14.4 (7)	C10—C9—N2—Co1	178.9 (3)
O1—C7—C8—C9	1.3 (7)	C12—C11—N2—C9	167.4 (4)
C6—C7—C8—C9	−176.5 (4)	C12—C11—N2—Co1	−11.1 (5)
C7—C8—C9—N2	−4.2 (7)	O1—Co1—N2—C9	9.8 (3)
C7—C8—C9—C10	173.5 (4)	N1—Co1—N2—C9	−170.1 (3)
N2—C11—C12—N1	8.4 (6)	O3—Co1—N2—C9	98.8 (3)
N2—C11—C12—C13	−171.7 (4)	N3—Co1—N2—C9	−81.8 (3)
N1—C12—C13—C14	−0.5 (7)	O1—Co1—N2—C11	−171.8 (3)
C11—C12—C13—C14	179.6 (4)	N1—Co1—N2—C11	8.3 (3)
C12—C13—C14—C15	−0.2 (7)	O3—Co1—N2—C11	−82.8 (3)
C13—C14—C15—C16	0.5 (7)	N3—Co1—N2—C11	96.6 (3)
C14—C15—C16—N1	0.0 (7)	O1—Co1—N3—N4	50.9 (4)
O3—C18—C19—C20	−0.4 (8)	N2—Co1—N3—N4	147.3 (4)
C17—C18—C19—C20	179.5 (4)	O2—Co1—N3—N4	−36.3 (4)
C18—C19—C20—O2	5.4 (8)	N1—Co1—N3—N4	−128.4 (4)
C18—C19—C20—C21	−171.3 (4)	C8—C7—O1—Co1	9.0 (6)
O2—C20—C21—C26	2.6 (6)	C6—C7—O1—Co1	−173.0 (3)
C19—C20—C21—C26	179.7 (4)	N2—Co1—O1—C7	−12.5 (3)
O2—C20—C21—C22	−173.9 (4)	O2—Co1—O1—C7	166.0 (3)
C19—C20—C21—C22	3.2 (6)	O3—Co1—O1—C7	−99.4 (3)
C26—C21—C22—C23	−2.8 (6)	N3—Co1—O1—C7	77.0 (3)
C20—C21—C22—C23	173.8 (4)	C19—C20—O2—Co1	−2.6 (6)
C21—C22—C23—C24	1.5 (7)	C21—C20—O2—Co1	174.4 (2)
C22—C23—C24—C25	−0.4 (8)	O1—Co1—O2—C20	86.7 (3)
C23—C24—C25—C26	0.6 (8)	N1—Co1—O2—C20	−93.4 (3)
C22—C21—C26—C25	3.0 (7)	O3—Co1—O2—C20	−2.5 (3)
C20—C21—C26—C25	−173.6 (4)	N3—Co1—O2—C20	178.4 (3)
C24—C25—C26—C21	−2.0 (8)	C19—C18—O3—Co1	−6.4 (6)
C13—C12—N1—C16	1.0 (6)	C17—C18—O3—Co1	173.6 (3)
C11—C12—N1—C16	−179.1 (4)	O1—Co1—O3—C18	−80.2 (3)
C13—C12—N1—Co1	177.9 (4)	N2—Co1—O3—C18	−176.7 (3)
C11—C12—N1—Co1	−2.2 (5)	O2—Co1—O3—C18	6.9 (3)
C15—C16—N1—C12	−0.7 (6)	N1—Co1—O3—C18	99.1 (3)

### Hydrogen-bond geometry (Å, °)

**Table:**

*D*—H···*A*	*D*—H	H···*A*	*D*···*A*	*D*—H···*A*
C1—H1···O1	0.93	2.38	2.699 (6)	100
C16—H16···O2	0.93	2.41	2.917 (6)	114
C26—H26···O2	0.93	2.34	2.661 (5)	100

## References

[bb1] Brandenburg, K. (1999). *DIAMOND.* Crystal Impact GbR, Bonn, Germany.

[bb2] Bruker (2007). *APEX2* and *SAINT* Bruker AXS Inc., Madison, Wisconsin, USA.

[bb3] Clearfield, A., Gopal, R., Kline, R. J., Sipski, M. & Urban, L. O. (1978). *J. Coord. Chem.* **7**, 163–169.

[bb4] Ray, A., Pilet, G., Gómez-García, C. J. & Mitra, S. (2009). *Polyhedron*, **28**, 511–520.

[bb5] Sheldrick, G. M. (1996). *SADABS. *University of Göttingen, Germany.

[bb6] Sheldrick, G. M. (2008). *Acta Cryst.* A**64**, 112–122. 10.1107/S0108767307043930 18156677

